# Novel long noncoding RNA OTUD6B-AS1 indicates poor prognosis and inhibits clear cell renal cell carcinoma proliferation via the Wnt/β-catenin signaling pathway

**DOI:** 10.1186/s12943-019-0942-1

**Published:** 2019-01-22

**Authors:** Gang Wang, Zi-jian Zhang, Wen-gang Jian, Pan-hong Liu, Wei Xue, Teng-da Wang, Yu-yang Meng, Chao Yuan, Hao-ming Li, Yi-peng Yu, Zhan-xin Liu, Qiong Wu, Da-ming Zhang, Cheng Zhang

**Affiliations:** 1 Department of Urology, The First Affiliated Hospital of Harbin Medical University, Harbin, 150001 Heilongjiang China; 2 Department of Cardiology, KaiFeng Central Hospital, KaiFeng, Henan Province China; 3 Department of Venous Injection Distribution Center, The First Affiliated Hospital of Harbin Medical University, Harbin, Heilongjiang Province China; 4 School of Life Science and Technology, Harbin Institute of Technology, Harbin, People’s Republic of China; 5 Department of Neurosurgery, The First Affiliated Hospital of Harbin Medical University, Harbin, 150001 Heilongjiang China

**Keywords:** Long noncoding RNA, OTUD6B-AS1, ccRCC, Proliferation, Wnt/β-catenin signaling

## Abstract

**Background:**

The long noncoding RNA (lncRNA) OTUD6B antisense RNA 1 (OTUD6B-AS1) is oriented in an antisense direction to the protein-coding gene OTUD6B on the opposite DNA strand. TCGA database data show that the expression of the lncRNA OTUD6B-AS1 is downregulated and that OTUD6B-AS1 acts as an antioncogene in a variety of tumors. However, the expression and biological functions of the lncRNA OTUD6B-AS1 are still unknown in tumors, including clear cell renal cell carcinoma (ccRCC).

**Methods:**

The expression level of OTUD6B-AS1 was measured in 75 paired human ccRCC tissue and corresponding adjacent normal renal tissue samples. The correlations between the OTUD6B-AS1 expression level and clinicopathological features were evaluated using the chi-square test. The effects of OTUD6B-AS1 on ccRCC cells were determined via MTT assay, clone formation assay, transwell assay, and flow cytometry. Furthermore, the impact of OTUD6B-AS1 overexpression on the activation of the Wnt/β-catenin signaling pathway was investigated. Finally, ACHN cells with OTUD6B-AS1 overexpression were subcutaneously injected into nude mice to evaluate the influence of OTUD6B-AS1 on tumor growth in vivo.

**Results:**

In this study, we found that the expression of the lncRNA OTUD6B-AS1 was downregulated in ccRCC tissue samples and that patients with low OTUD6B-AS1 expression had shorter overall survival than patients with high OTUD6B-AS1 expression, which showed that the different expression level of OTUD6B-AS1 indirectly correlated with survival of patients. Lentivirus-mediated OTUD6B-AS1 overexpression significantly decreased the proliferation of ccRCC cells and promoted the apoptosis of the cells. Furthermore, OTUD6B-AS1 overexpression partly inhibited cell migration and invasion. The overexpression of OTUD6B-AS1 decreased the activity of the Wnt/β-catenin pathway and suppressed the expression of epithelial-to-mesenchymal transition (EMT)-related proteins (E-cadherin, N-cadherin and Snail) in ccRCC cells. In addition, compared with the parental ACHN cells, OTUD6B-AS1-overexpressing ACHN cells injected into nude mice exhibited decreased tumor growth in vivo.

**Conclusions:**

Taken together, our findings present a road map for targeting the newly identified lncRNA OTUD6B-AS1 to suppress ccRCC progression in cell lines, and these results elucidate a novel potential therapeutic target for ccRCC treatment.

**Electronic supplementary material:**

The online version of this article (10.1186/s12943-019-0942-1) contains supplementary material, which is available to authorized users.

## Introduction

Renal cell carcinoma (RCC) is a highly malignant tumor of the urinary system. It is also one of the most common clinical types [1]. It is a malignant tumor that originates in the renal parenchyma urinary tubule epithelial system. The incidence of RCC is third among urological tumors, second to only prostate and bladder cancers [2]. It accounts for 3% of adult malignancies and 80–90% of renal tumors, of which the male-to-female patient ratio is approximately 2:1 [3]. Regarding the pathological types of RCC, approximately 75–80% of cases are clear cell renal cell carcinoma (ccRCC) [4, 5]. Because nearly 1/3 of patients have localized or distant metastasis at the initial diagnosis and because ccRCC is not sensitive to radiotherapy or chemotherapy but is treatable by surgery, although the recurrence rate is still up to 20–40% after radical nephrectomy, so the 5-year survival rate of ccRCC is only approximately 20% after surgical resection [6, 7]. Therefore, finding new targets against the proliferation and metastasis of ccRCC cells is urgently required.

LncRNAs are eukaryotic cell genome-encoded transcripts larger than 200 RNA nucleotides that lack obvious open reading frames and do not encode proteins; thus, they are called long noncoding RNAs [8]. Although lncRNAs do not participate in the encoding of protein, they can regulate gene expression at different levels, such as the epigenetic, transcriptional and posttranscriptional levels, and play key roles in genome modification, transcriptional activation, transcriptional interference, and chromosome sedimentation [9]. Several studies have confirmed that abnormal expression of lncRNAs is closely related to the biological behaviors of malignant tumor cells, such as growth, proliferation, invasion and metastasis [10]. For instance, the lncRNA HOTAIR is highly expressed in a variety of cancer cells and regulates the methylation level of histone H3K27 by recruiting PRC2 to specific locus, leading to the formation of repressor chromatin complexes and the epigenetic silencing of tumor suppressor genes, which thus promotes the occurrence and progression of multiple malignancies, including colorectal cancer, cervical cancer, and RCC [11, 12]. High expression of MEG3 can regulate the level of histone H3 methylation by recruiting the histone methyltransferase EZH2 and the histone demethylation enzyme JARID2 to the regulatory regions of the epithelial marker gene CDH1 and microRNA-200 family genes, which play a key role in the EMT process mediated by TGF-beta, which ultimately results in lung cancer and regulates EMT in lung cancer at the epigenetic level. In addition, high expression of MEG3 is closely related to the malignancy of hepatocellular carcinoma, gastric cancer, colorectal cancer and bladder cancer [13]. In addition, a number of studies have confirmed that abnormal expression or functional changes in lncRNA expression are closely related to the formation, local progression, and distant metastasis of urinary tract tumors, such as prostate cancer [14], bladder cancer [15] and kidney cancer [16].

As for our research, we are the first to report the expression pattern, biological function and potential regulatory mechanism of OTUD6B-AS1 in ccRCC. Quantitative real-time PCR (qRT-PCR) assays revealed that OTUD6B-AS1 expression was significantly decreased in ccRCC tissue samples and cell lines, suggesting antioncogene functions for OTUD6B-AS1. The probable relationship between OTUD6B-AS1 and overall survival in ccRCC patients were also determined. Furthermore, our findings indicated that the lncRNA OTUD6B-AS1 inhibited ccRCC proliferation through the inactivation of the Wnt/β-catenin pathway and suppressed the expression of EMT-related proteins.

## Materials and methods

### Patient and clinical information

A total of 75 paired ccRCC tissue and adjacent normal tissue samples were collected from ccRCC patients who underwent radical nephrectomy at the First Affiliated Hospital of Harbin Medical University. All the samples were immediately snap frozen in liquid nitrogen for long-term preservation until RNA extraction.

### Cell lines and cell culture

The human ccRCC cell lines (786-O, Caki-1, 769-P, OS-RC-2, and ACHN) and human renal tubular epithelial cells (HK-2) were all purchased from the Cell Resources Center, Shanghai Academy of Life Sciences, Chinese Academy of Sciences. ACHN cells were grown in RPMI-DMEM (KeyGen, Nanjing, China); HK-2, 786-O, Caki-1, 769-P, and OS-RC-2 cells were grown in RPMI 1640 medium (KeyGen, Nanjing, China) containing 10% FBS (Life Technologies, Australia). All cells were grown at 37 °C in a humidified 5% CO_2_ atmosphere.

### Cell transfection

A plvx-OTUD6B-AS1 vector and an empty plvx-vector (vector) were commercially synthesized by GenePharma (Shanghai, China). For transient transfection, ACHN and OS-RC-2 cells were plated into six-well plates (2 × 10^5^/well) and routinely maintained for 24 h at 37 °C. Then, the cells were transfected with plvx-OTUD6B-AS1 or the empty plvx-vector according to the manufacturer’s protocol. Subsequent experiments were performed at 48 h posttransfection.

### Treatment of ACHN and OS-RC-2 cells with 5-aza-2-deoxycytidine

ACHN and OS-RC-2 cells were seeded into 6-well plates at 3 × 10^5^ cells per well and cultured in RPMI-DMEM or RPMI 1640 medium containing 5 μM 5-aza-2-deoxycytidine (5-Aza-CdR, Sigma-Aldrich, USA) for 5 days according to a previously reported protocol [17, 18]. The cells treated with 5-Aza-CdR were harvested and used for detection in plvx-OTUD6B-AS1 expression assays.

### Cell proliferation assay

Cell proliferation was assayed using an MTT assay. The transfected cells were plated into 96-well plates (5000 cells/well). Cell proliferation was detected every 24 h according to the manufacturer’s protocol. Briefly, 20 μl of MTT solution was added to each well and incubated for 4 h at 37 °C. The solution was then discarded, and 150 μl of DMSO was added. After shaking for 15 min at room temperature, the absorbance of the solution was measured with a spectrophotometer at 490 nm.

### Total RNA extraction and quantitative real-time PCR

Total RNA was extracted from the ccRCC tissue samples and cell lines using TRIzol reagent (Ambion, Life Technologies, USA) according to the manufacturer’s instructions. Total RNA was reverse transcribed into cDNA using a First Strand cDNA Synthesis Kit (TOYOBO Life Science, Shanghai, China) for the detection of OTUD6B-AS1 expression. The relative expression of OTUD6B-AS1 was detected using FastStart Universal SYBR Green Master Mix (ROX) and normalized to that of GAPDH. The primer sequences were as follows: 5’-GACATATCCGGGTGACGTTTTT-3′ (sense) and 5’-TTGTTCCACTGTCTTCTGGCATT-3′(antisense) for OTUD6B-AS1 and 5’-CACCCACTCCTCCACCTTTGA-3′ (sense) and 5’-ACCACCCTGTTGCTGTAGCCA-3′ (antisense) for GAPDH. The results were analyzed using the -ΔCt method. All the results are expressed as the mean ± SD of three independent experiments.

### Colony formation assay

ACHN and OS-RC-2 cells (0.1 × 10^3^ cells per well) were seeded in a six-well plate and cultured for 10 days after treatment. Colonies were then fixed with 4% paraformaldehyde for 15 min and stained for 10 min with 0.5% crystal violet. Then, the number of colonies was counted using ImageJ, and images were taken under an Olympus microscope (Tokyo, Japan).

### Cell immunofluorescence staining

Treated cells were fixed in 4% paraformaldehyde for 30 min, and then 0.5% Triton X-100 was used to permeabilize the cells at room temperature for 20 min. After blocking with 5% BSA, slides were incubated overnight at 4 °C with a primary antibody against ki-67 (1:1000, Abcam, Cambridge, UK). Then, the slides were incubated with a fluorescent secondary antibody for 1 h in a wet box at room temperature in the dark. Finally, DAPI was used to counterstain the nuclei. Images were taken under an Olympus microscope (Tokyo, Japan).

### Hoechst staining

After transfection, cells were fixed for 10 min with a fixation solution and washed twice with phosphate-buffered saline (PBS) for 3 min each time, and then the liquid was drained. Finally, 0.5 ml of Hoechst staining solution (Wanleibio, Shenyang, China) was added for 5 min to dye the cells. Images were taken under an Olympus microscope (Tokyo, Japan). It can be seen that the nuclei of apoptotic cells are densely stained or fragmented densely stained. Cyan represents cells that have undergone apoptosis.

### Flow cytometry analysis of apoptosis and cell cycling

After transfection, the apoptosis rates of ACHN and OS-RC-2 cells were analyzed using flow cytometry. The cells were collected and washed twice with cold PBS. Subsequently, the cells were stained with an anti-Annexin V-PE antibody and 7-AAD dye for 15 min in the dark according to the manufacturer’s instructions. The stained cells were analyzed with a flow cytometer (BD Biosciences) and were counted using CellQuest software (BD Biosciences). After staining with propidium iodide (PI) using a CycleTESTTM PLUS DNA Reagent Kit (BD Biosciences), a flow cytometer (FACScan®; BD Biosciences) was used to analyze the cells. The cell cycle results elucidate the exact distribution of the cells in the G0-G1, S, and G2-M phases. This experiment was repeated three times.

### Western blot analysis

Total protein was prepared from ACHN and OS-RC-2 cells using RIPA buffer with a proteinase inhibitor. The lysates were incubated on ice for 30 min and then centrifuged at 12,000 rpm for 15 min at 4 °C, and the protein concentration was measured by a BCA kit (Beyotime Biotechnology, Beijing, China). Equal quantities of protein were electrophoresed through a 12.5% sodium dodecyl sulfate-polyacrylamide gel and transferred to nitrocellulose membranes (Millipore, Billerica, MA). The membranes were blocked and then incubated with the appropriate primary antibody and β-actin overnight at 4 °C. Subsequently, the membranes were incubated with an anti-mouse or anti-rabbit secondary antibody (Santa Cruz Biotechnology) at room temperature for 1 h. The protein bands were visualized using a chemiluminescence reagent (ECL) kit (Beyotime Biotechnology).

### Immunohistochemistry (IHC)

Tumor tissues from mice were fixed in 4% paraformaldehyde, dehydrated, embedded in paraffin, and cut into 4-μm-thick sections. The sections were deparaffinized, blocked against endogenous peroxidase activity and underwent antigen retrieval. After blocking with 5% bovine serum albumin, the sections were incubated overnight at 4 °C with a primary antibody against ki-67 (1:500, Abcam, Cambridge, UK). Then, the sections were incubated with a peroxidase-conjugated polymer for 30 min, and a DAB (Beyotime Institute of Biotechnology, Jiangsu, China) system was used for detection.

### In vivo tumor formation assay

Female nude mice that were 4 weeks old were supplied by Beijing Vital River Laboratory Animal Technology Co., Ltd. (Beijing, China). ACHN cells stably transfected with the empty vector or plvx-OTUD6B-AS1 were collected, and the concentration of resuspended ACHN cells was 2 × 10^7^ cells/mL. Then, the suspended cells were injected into either side of the posterior flank of each mouse. Tumor volumes were tested every 3 days. The mice survived for up to 34 days after the injection before they needed to be sacrificed. Then, the tumors formed from the empty-vector-transfected or plvx-OTUD6B-AS1-transfected ACHN cells were removed from the mice and kept for weight measurement, immunohistochemistry (IHC) and hematoxylin-eosin staining (H&E). The protocol conformed with the regulations of Harbin Medical University’s Animal Ethics Committee.

### Statistical analysis

Statistical data were analyzed using Statistical Program for Social Sciences (SPSS) 19.0 software (SPSS, Chicago, IL, USA). GraphPad Prism 7.0 (GraphPad Software, La Jolla, CA) was used to plot all graphs. The differences between the tumor tissue and normal colonic mucosa were analyzed by a paired t-test. A receiver operating characteristic (ROC) curve was constructed to evaluate the diagnostic values. *P* < 0.05 was considered statistically significant.

## Results

### Comprehensive analysis of the lncRNA OTUD6B-AS1 in the TCGA database

**Fig. 1:**
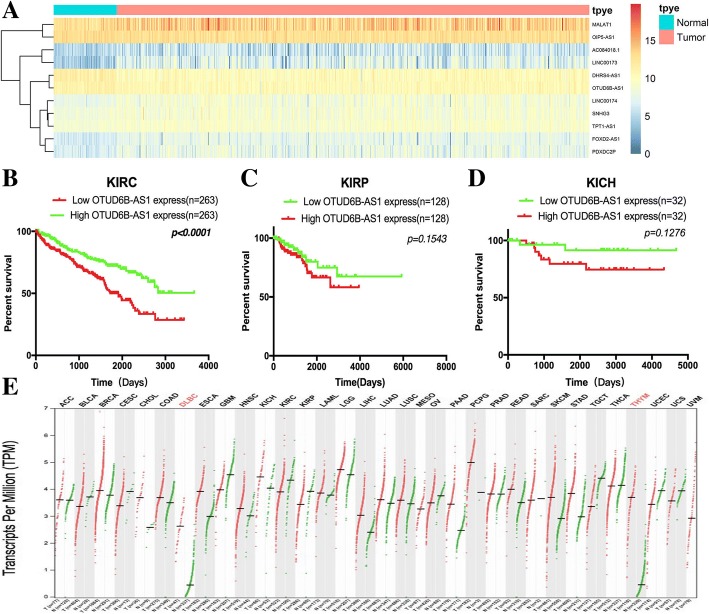
Comprehensive analysis of the lncRNA OTUD6B-AS1 in the TCGA database. a The heatmap of ten lncRNA expression profiles. (b, c, d) Kaplan-Meier plots of the KIRC, KIRP and KICH cohorts

Data for 526 TCGA ccRCC patients were downloaded. Based on the set, the lncRNAs were subjected to a univariate Cox regression model, and a total of 23 lncRNAs were significantly correlated with patient overall survival (*p*-value < 0.05; Fig. 1a, Additional file 1: Table S1). Then, the other integrated data from 256 kidney papillary (KIRP) and 64 kidney chromophobe (KICH) patients in the TCGA database were enrolled in the analyses. Demographic, clinical, follow-up and tumor pathology data for the three RCC subtypes are listed in Additional file 1: Table S2.

In the kidney clear cell carcinoma (KIRC) cohort, the patients in the high OTUD6B-AS1 expression group had a five-year survival rate 22.8% higher than the five-year survival rate of 15.2% of the patients in the low OTUD6B-AS1 expression group. The overall survival status significantly differed between the high OTUD6B-AS1 expression group and the low OTUD6B-AS1 expression group (HR = 1.962, 95% CI 1.445–2.664, *p* < 0.0001; Fig. 1b). However, no significant difference in overall survival status was found between the high OTUD6B-AS1 expression group and the low OTUD6B-AS1 expression group in the KIRP and KICH cohorts (Fig. 1c and d).

Then, Kaplan-Meier analysis was used to evaluate the relationship between OTUD6B-AS1 expression in ccRCC and patient clinicopathological characteristics, and the results showed that the survival time of the patients with pathologic stage I + II disease (*n* = 318) was longer than that of the patients with advanced stage lesions (*n* = 205) (*P* < 0.0001, Additional file 1: Figure S1A). The survival time of the patients with clinical grade G1 + G2 disease (*n* = 238) was longer than that of the patients with advanced stage lesions (*n* = 278) (*P* < 0.0001, Additional file 1: Figure S1B).

In order to understand the expression of A in different tumors, we extracted A from TCGA database in all 33 tumors and found that A was low in most tumors (Fig. 1e).

The expression of the lncRNA OTUD6B-AS1 was downregulated in ccRCC tissue compared with normal tissue and was associated with clinicopathological features of patients with ccRCC.

**Fig. 2:**
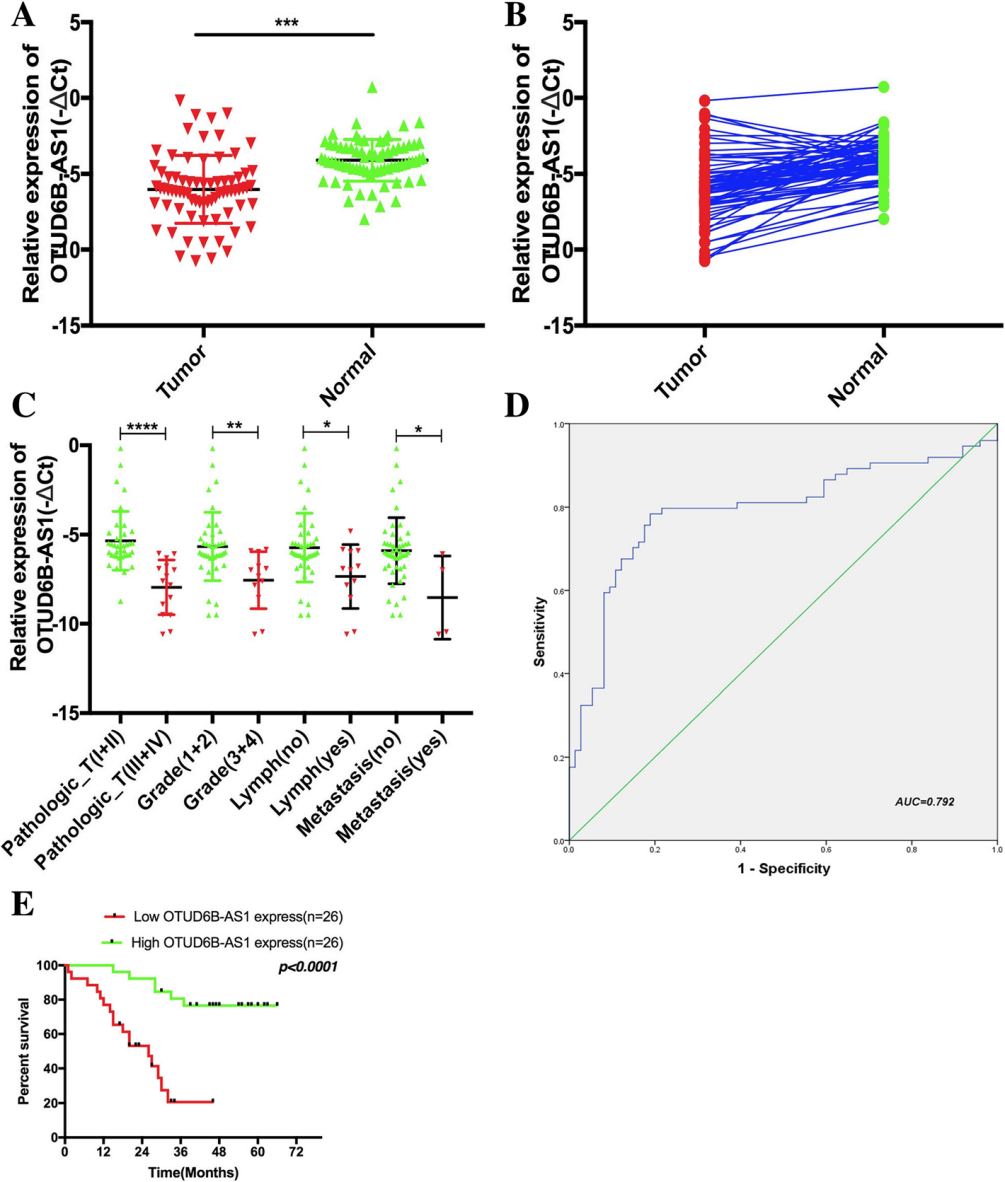
LncRNA OTUD6B-AS1 expression was downregulated in ccRCC tissue and associated with clinicopathological features of patients with ccRCC. (a, b) The relative expression of OTUD6B-AS1 in ccRCC tissue (n = 75) compared with corresponding adjacent normal tissue (*n* = 75) is shown. OTUD6B-AS1 expression was examined by qRT-PCR and normalized to GAPDH expression (shown as the -ΔCT method). c The expression levels of OTUD6B-AS1 were determined by qRT-PCR in different clinical stage ccRCC tissue samples. d The ROC curve was constructed using SPSS. The area under the curve was 0.792. The sensitivity and specificity were 0.773 and 0.813, respectively. e The survival time of the patients with high OTUD6B-AS1 expression (*n* = 26) was longer than that of the patients with low OTUD6B-AS1 expression (*n* = 26). The data represent the mean ± SD of 3 replicates. * *P* < 0.05; ** *P* < 0.01; *** *P* < 0.001; **** *P* < 0.0001

To explore the role of the lncRNA OTUD6B-AS1 in ccRCC, we analyzed the expression of OTUD6B-AS1 in a cohort of 75 ccRCC tissue samples and matched nontumor samples using qRT-PCR analysis, with our results showing that OTUD6B-AS1 expression was remarkably decreased in the ccRCC tissue samples compared with the paired adjacent normal tissue samples (Fig. 2a and b). We also found that the expression of OTUD6B-AS1 was related to the pathologic tumor stage, clinical grade, lymph node status and metastasis status but not the gender and age of patients (Fig. 2c and Table 1). The same results were also verified with the TCGA database data (Additional file 1: Table S3). Then, we included the variables age, gender, clinical tumor grade, pathologic stage, lymph node status, metastasis status, and OTUD6B-AS1 mRNA level in a multivariate Cox regression model and found that the OTUD6B-AS1 mRNA level was an independent predictor for overall survival status in KIRC patients (HR = 0.275, 95% CI 0.081–0.930, *p* = 0.038; Table 2). The same results were also verified with the TCGA database data (Additional file 1: Table S4).

**Table 1:** The relationship of lncRNA OTUD6B-AS1 expression levels (-ΔCt) in cancer tissue with clinical features in KIRC patients

Variable	KIRC	Group		χ2	*p*-Value
		Low lncRNA	High lncRNA		
Sample (*n*)	52	26	26		–
Median circ	- - −6.0999141 (− 0.169--10.594333)	–	–		–
Median age (year)	56.8(35–82)	–	–	0.693	0.405
≤ 56	27(51.9%)	12	15		
> 56	25(48.1%)	14	11		
Gender				0.080	0.777
Male	31(59.6%)	15	16		
Female	21(40.4%)	11	10		
Clinical stage				5.026	0.025*
Stage I+ II	36(69.2%)	12	24		
Stage III+ IV	16(38.8%)	14	2		
Tumor stage				13.000	0.000*
T1+ T2	39(75.0%)	16	23		
T3+ T4	13(25.5%)	10	3		
Lymph node metastasis				4.591	0.032*
N0	37(71.6%)	15	22		
N1	15(28.4%)	11	4		
NX	–	–	–		
Distant metastasis				4.127	0.042*
M0	45(86.5%)	20	25		
M1	7(13.5%)	6	1		
MX	–	–	–		
Staus					0.002*
Dead	25(48.1%)	17	6	9.433	
Alive	27(51.9)	9	20		

Low/high by the sample mean. Pearson χ2-test. p < 0.05 was considered statistically significant

**Table 2:** Univariable and multivariable Cox regression analyses in KIRC

Characteristics	Subset	Univariate analysis	*P* value	Multivariate analysis	*P* value
		Hazard ratio(95% CI)		Hazard ratio(95% CI)	
Age	≤ 56/> 56	1.849(0.806–4.241)	0.147	1.284(0.479–3.443)	0.620
Gender	Male/Female	1.006(0.434–2.330)	0.990	1.332 (0.463–3.831)	0.595
Pathologic_T	I + II/III + IV	11.318(4.498–28.478)	0.000^*^	4.589(1.151–18.294)	0.031^*^
Grade	Grade(1 + 2)/Grade(3 + 4)	13.919(5.289–36.631)	0.000^*^	6.439(1.060–39.133)	0.043^*^
Lymph node metastasis	Yes/No	0.122(0.051–0.294)	0.000^*^	0.753 (0.110–5.145)	0.772
Distant metastasis	Yes/No	0.240(0.093–0.621)	0.003^*^	0.995(0.283–3.493)	0.995
Expression	High/Low	0.145(0.054–0.391)	0.000^*^	0.275 (0.081–0.930)	0.038^*^

In both univariable and multivariable Cox regression analyses, age, gender, Pathologic_T and Tumor grade, Lymph node metastasis, Distant metastasis, Expression were evaluated as continuous variables. P < 0.05 was considered statistically significant in all analyses.

To assess whether OTUD6B-AS1 could be used as an indicator for the diagnosis of ccRCC, an ROC curve was built. A total of 75 patient normal kidney tissue samples were used as controls to produce this ROC curve (Fig. 2d). The sensitivity and specificity were 0.773 and 0.814, respectively. The cutoff value was − 4.866. The area under the curve was 0.792 (95% CI = 0.715–0.870, *P* < 0.000). The Youden index was 0.586. Therefore, OTUD6B-AS1 could be used as an indicator of ccRCC.

Kaplan-Meier analysis was used to evaluate the relationship between OTUD6B-AS1 expression in ccRCC and patient survival, and the results showed that lower OTUD6B-AS1 expression was associated with poor survival. The survival time of the patients with high OTUD6B-AS1 expression (*n* = 26) was longer than that of the patients with low OTUD6B-AS1 expression (*n* = 26) (*P* < 0.0001, Fig. 2e). The survival time of the patients with pathology stage I + II (*n* = 36) and clinical grade I + II (*n* = 39) disease was longer than that of the patients with advanced stage (*n* = 16) and grade (*n* = 13) lesions (*P* < 0.0001, Additional file 1: Figure S1C and D).

### LncRNA OTUD6B-AS1 was downregulated in ccRCC cell lines

**Fig. 3:**
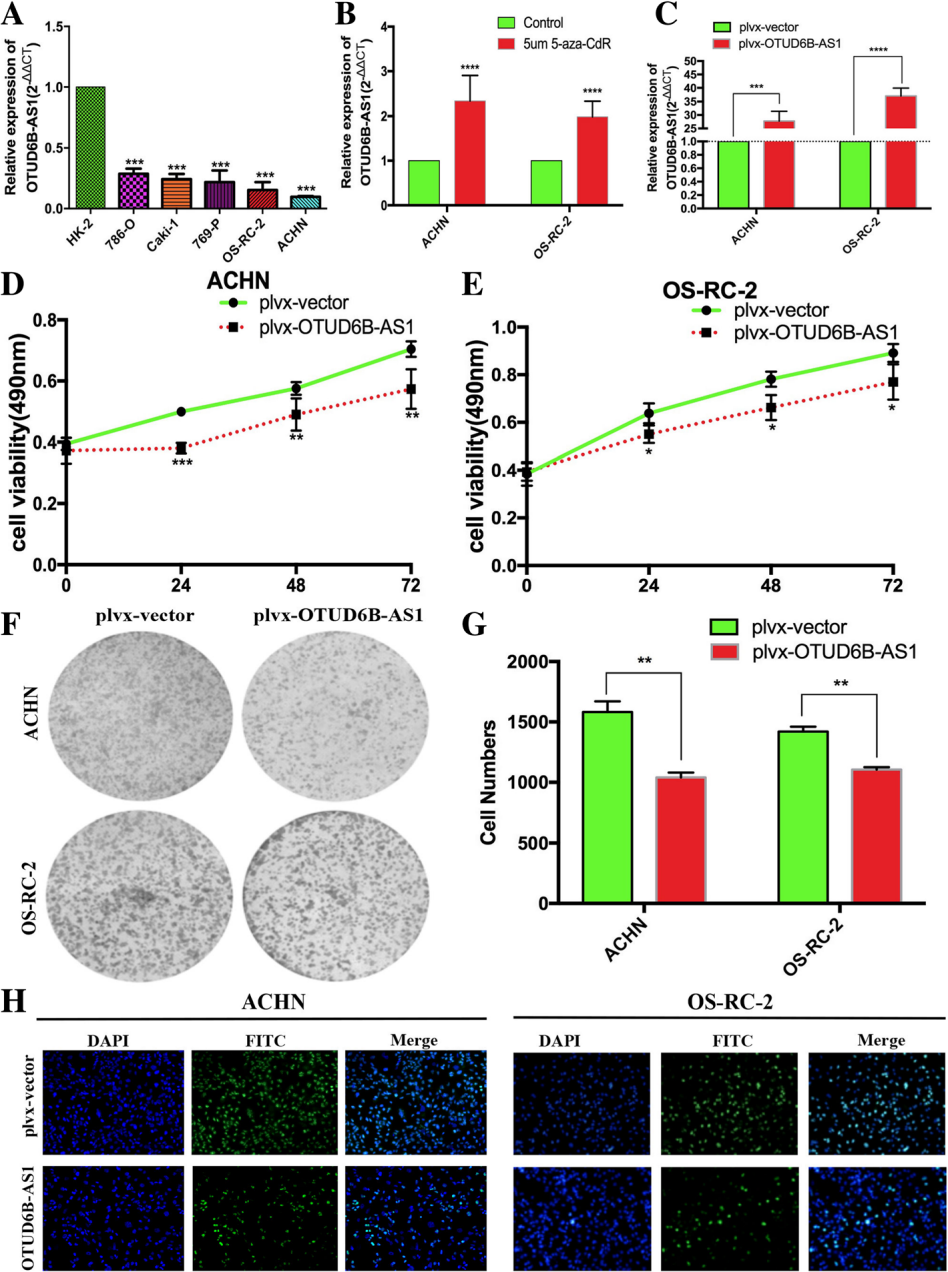
Overexpression of OTUD6B-AS1 markedly suppressed the proliferation of ccRCC cells in vitro. a OTUD6B-AS1 expression levels in the ccRCC cell lines (786-O, Caki-1769-P, OS-RC-2 and ACHN) compared with that in the human renal tubular epithelial cell line (HK-2). b ACHN and OS-RC-2 cells treated with 5 μM 5-aza-CdR. c qRT-PCR analysis of the OTUD6B-AS1 expression in the ACHN and OS-RC-2 cells transfected with plvx-OTUD6B-AS1 or the empty vector. (d, e) MTT cell proliferation assays performed with the ACHN and OS-RC-2 cells transfected with plvx-OTUD6B-AS1 or the empty vector. (f, g) Colony formation assays performed with the ACHN and OS-RC-2 cells transfected with plvx-OTUD6B-AS1 or the empty vector. h Cell immunofluorescence staining assay performed with the ACHN and OS-RC-2 cells transfected with plvx-OTUD6B-AS1 or the empty vector. *P < 0.05, **P < 0.01, *** *P* < 0.001

To test the OTUD6B-AS1 expression levels in ccRCC cells, we performed qRT-PCR assays and found that the expression levels of OTUD6B-AS1 were downregulated in the ccRCC cell lines compared with HK-2 cells. In this study, we selected ACHN and OS-RC-2 cells as they had the lowest OTUD6B-AS1 expression among the ccRCC cell lines (Fig. 3a). In this section, we evaluated the effect of a DNA demethylating agent (5-Aza-CdR) on OTUD6B-AS1 expression at the cellular level. First, we found that the OTUD6B-AS1 promoter was methylated by consulting the UCSC database (http://genome.ucsc.edu). Following the treatment of ACHN and OS-RC-2 cells with 5-Aza-CdR, the expression level of OTUD6B-AS1 was significantly higher in the 5-Aza-CdR-treated cells than in the control cells (Fig. 3b). Then, OTUD6B-AS1 was overexpressed in ACHN and OS-RC-2 cells transfected with the plvx-OTUD6B-AS1. qRT-PCR analysis was performed at 48 h posttransfection, and the data revealed that OTUD6B-AS1 expression was significantly increased by plvx-OTUD6B-AS1 compared with the empty vector. (Fig. 3c).

### Overexpression of OTUD6B-AS1 markedly suppressed the proliferation of ccRCC cells in vitro

To identify the function of OTUD6B-AS1 in ccRCC, we performed gain-of-function assays. MTT assays showed that the growth of the ACHN and OS-RC-2 cells transfected with plvx-OTUD6B-AS1 was inhibited relative to that of the control cells (Fig. 3d and e). Similarly, increased OTUD6B-AS1 expression impaired the colony formation capacities of ccRCC cells (Fig. 3f and g). These findings were confirmed by the results of ki-67 staining assays (Fig. 3h) and highlighted OTUD6B-AS1 as an antioncogene in ccRCC cells.

### OTUD6B-AS1 overexpression inhibited the migration and invasion of ccRCC cells in vitro

**Fig. 4:**
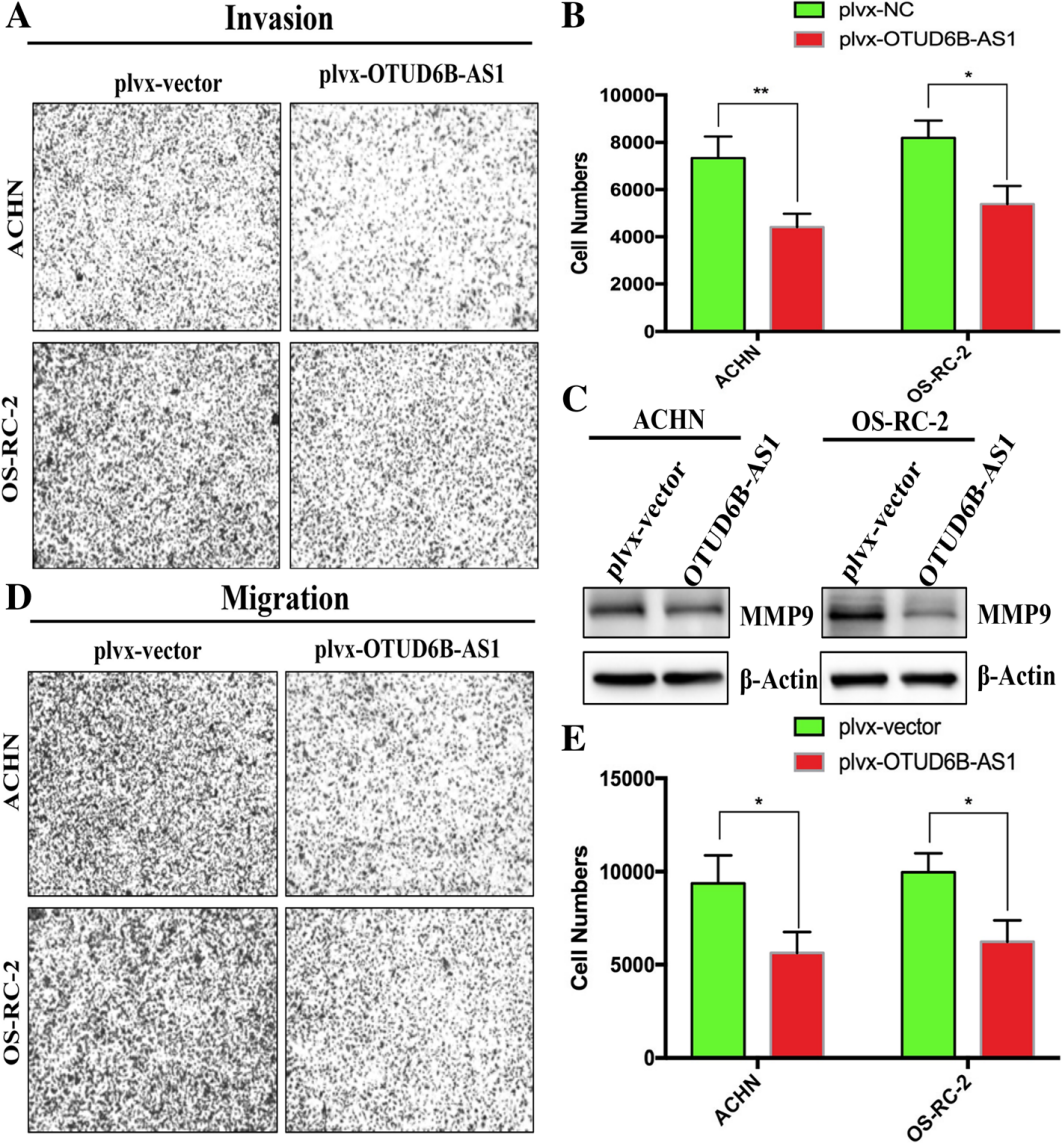
OTUD6B-AS1 overexpression inhibited the migration and invasion of ccRCC cells in vitro. (a, b) Transwell assays with Matrigel for cell invasion with the ACHN and OS-RC-2 cell lines transfected with plvx-OTUD6B-AS1 or the empty vector; c western blot analysis of MMP9 expression in cells transfected with plvx-OTUD6B-AS1 or the empty vector. β-Actin internal control. d, e Transwell assays without Matrigel for cell migration with the ACHN and OS-RC-2 cell lines transfected with plvx-OTUD6B-AS1 or the empty vector. The data represent the mean ± SD of 3 replicates. *Indicates a significant difference between the empty vector group and the plvx-OTUD6B-AS1 group. * *P* < 0.05, **P < 0.01

Next, we studied whether OTUD6B-AS1 could affect the migration and invasion of ccRCC cells. Directional invasion was examined using a transwell assay with Matrigel-coated upper compartments. The results showed that the invasion of ACHN (upper) and OS-RC-2 (lower) cells was notably decreased with OTUD6B-AS1 overexpression (Fig. 4a and b). In addition, the expression level of the invasion-related gene MMP9 was correspondingly decreased (Fig. 4c). Furthermore, we investigated the effect of OTUD6B-AS1 on cell migration by performing a transwell assay without a Matrigel coating in the upper compartment. Compared with the cells transfected with the control plvx-vector, the OTUD6B-AS1-overexpressing cells exhibited attenuated migratory abilities(Fig. 4d and e).

### Upregulation of OTUD6B-AS1 expression induces ccRCC cell apoptosis and alters cell cycle progression in vitro

**Fig. 5:**
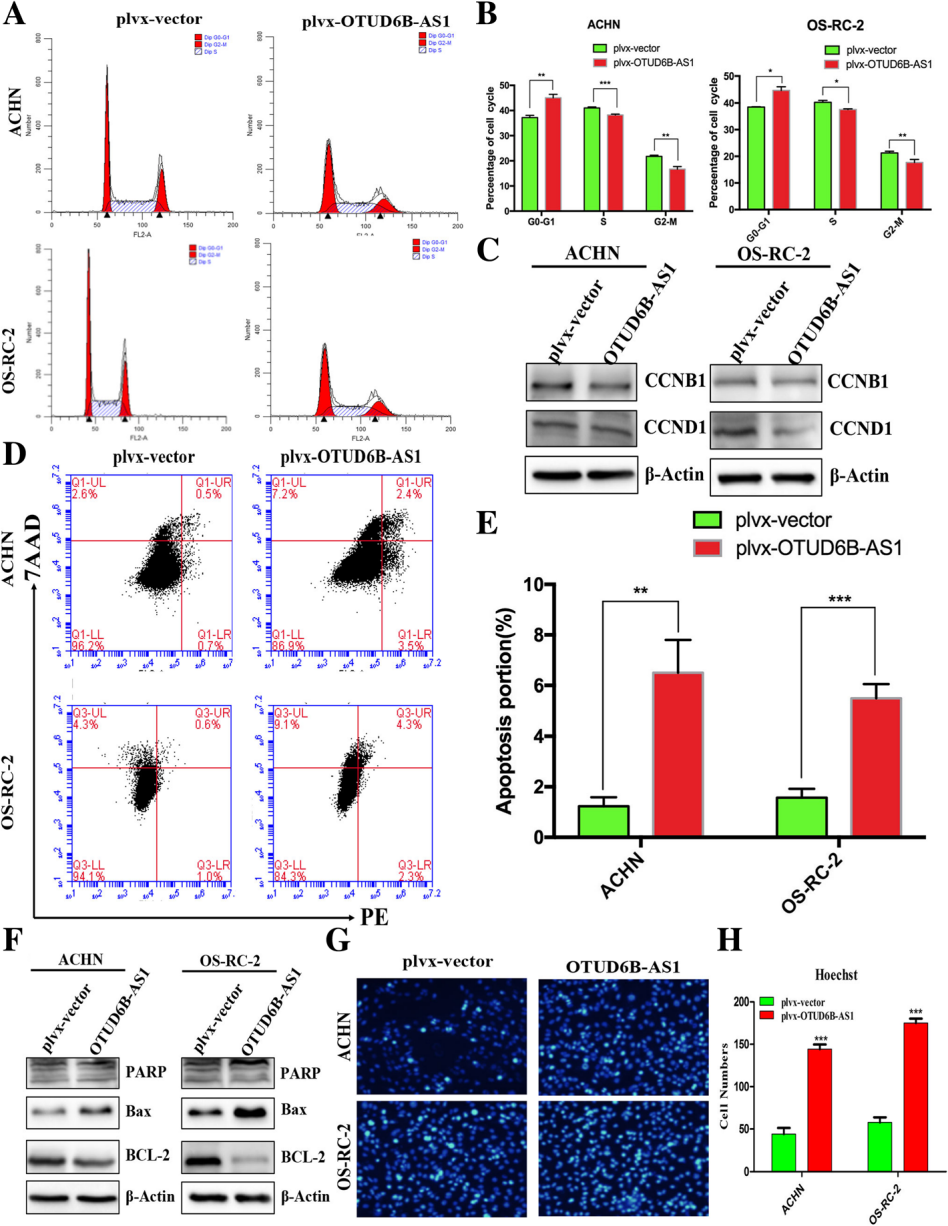
Upregulation of OTUD6B-AS1 expression induced ccRCC cell apoptosis and altered cell cycle progression in vitro. a, b Flow cytometry assays were performed to analyze the cell cycle progression of the ACHN and OS-RC-2 cell lines transfected with plvx-OTUD6B-AS1 or the empty vector. The bar chart represents the percentages of cells in the G0/G1, S, or G2/M phase as indicated. All experiments were performed in biological triplicates with three technical replicates. c Western blot analysis was performed to assess the expression of CCNB1 and CCND1 in cells transfected with plvx-OTUD6B-AS1 or the empty vector. GAPDH protein was used as an internal control. d, e Flow cytometry was used to detect the apoptosis rates of the cells. LR, early apoptotic cells; UR, terminal apoptotic cells. f Western blot analysis was used to measure the levels of PARP, Bax and Bcl-2 in cells transfected with plvx-OTUD6B-AS1 or empty vector. β-Actin protein was used as an internal control. g Hoechst staining assay performed in ACHN and OS-RC-2 cells transfected with plvx-OTUD6B-AS1 or the empty vector. The nuclei of the apoptotic cells were dense or dyed (Cyan)

Cell proliferation is regulated by the cell cycle and apoptosis. To evaluate the impact of OTUD6B-AS1 on the ccRCC cell cycle and apoptosis, flow cytometry assays and Hoechst staining analysis were conducted. We observed a significant increase in the quantity of cells in the G0/G1 phase and a decrease in the quantity of cells in the G2/M phase in the ACHN and OS-RC-2 cells transfected with plvx-OTUD6B-AS1 compared with the respective control cells (Fig. 5a and b). Additionally, western blot assays revealed remarkable alterations in CCNB1 and CCND1 expressing in the ACHN and OS-RC-2 cells overexpressing OTUD6B-AS1, confirming that OTUD6B-AS1 is involved in cell cycle regulation (Fig. 5c). Moreover, the ACHN and OS-RC-2 cells transfected with the OTUD6B-AS1 overexpression plasmid exhibited higher levels of apoptosis than the corresponding control cells (Fig. 5d and e). Furthermore, the protein expression levels of cleaved PARP and Bax exhibited significant increases, while Bcl-2 gene expression exhibited significant decreases in the ACHN and OS-RC-2 transfected with the OTUD6B-AS1 overexpression plasmid (Fig. 5f). Hoechst staining assays further confirmed these results (Fig. 5g and h). These data suggest that OTUD6B-AS1 overexpression can promote G1/G0 arrest, inhibit G2/M phase and increase the apoptotic rate of ccRCC cells in vitro.

### Overexpression of OTUD6B-AS1 decreased the activity of the Wnt/β-catenin pathway and suppressed the expression of EMT-related proteins

**Fig. 6:**
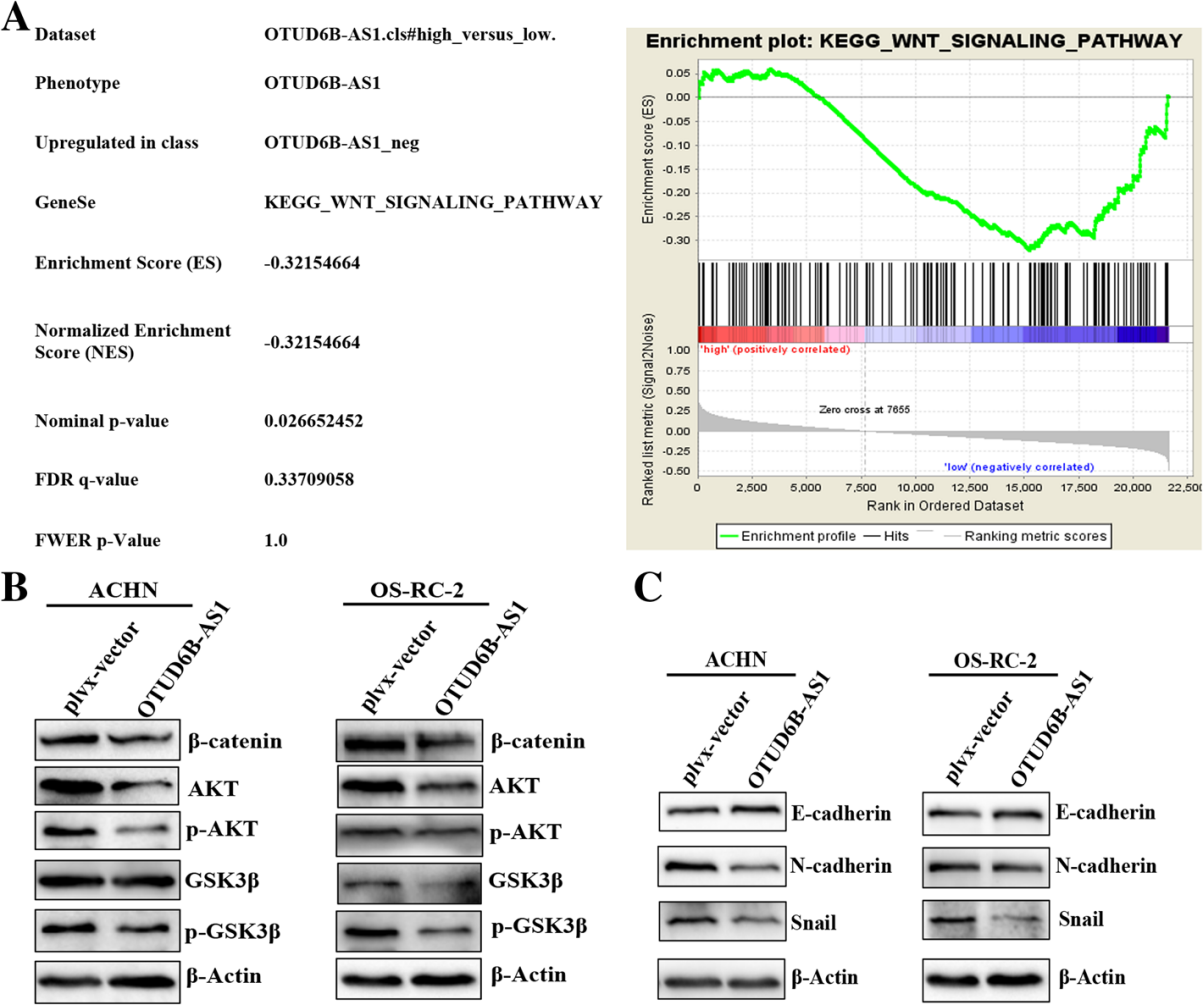
Overexpression of OTUD6B-AS1 decreased the activity of the Wnt/β-catenin pathway and suppressed the expression of EMT-related proteins. a Kyoto Encyclopedia of Genes and Genomes analysis revealed that the Wnt signaling pathway was significantly altered in ccRCC. b The effect of OTUD6B-AS1 on the Wnt/β-catenin signaling pathway was analyzed by western blotting with the indicated antibodies and samples from the ACHN and OS-RC-2 cells transfected with plvx-OTUD6B-AS1 or the empty vector. c The effect of OTUD6B-AS1 on EMT-related proteins was analyzed by western blotting with the indicated antibodies and samples from the ACHN and OS-RC-2 cells transfected with plvx-OTUD6B-AS1 or the empty vector

We investigated the possible pathways that contribute to the effects of OTUD6B-AS1 on ccRCC. The GSEA analyses of 72 ccRCC cases in the GSE53757 dataset of the GEO database confirmed the differences in cell processes between the two groups in the ccRCC cohort (Fig. 6a, Additional file 1: Table S5). Notably, the Wnt/β-catenin pathway was significantly altered in ccRCC. We investigated the consequences of OTUD6B-AS1 overexpression on the activation of the Wnt/β-catenin pathway in ccRCC cells. To test the effect of OTUD6B-AS1 overexpression on the Wnt/β-catenin signaling pathway, we analyzed the expression of several key proteins of this signaling pathway by western blotting. Compared with the control group, the OTUD6B-AS1 overexpression group exhibited decreases in both phosphorylated and nonphosphorylated AKT and glycogen synthase kinase 3β (GSK3β), and the total level of β-catenin was also decreased in both ACHN and OS-RC-2 cells (Fig. 6b). Additionally, to demonstrate the mechanism of OTUD6B-AS1 acting as an antioncogene in ccRCC cells, the expression of EMT-related proteins was determined by western blotting. The upregulation of OTUD6B-AS1 expression significantly upregulated the expression of the epithelial marker E-cadherin, while the expression levels of the mesenchymal cell markers N-cadherin and Snail were downregulated in both the ACHN and OS-RC-2 cells with enhanced expression of OTUD6B-AS1 (Fig. 6c). However, when we knocked down OTUD6B-AS1 expression in 786-O cells, we observed the opposite result. Based on the results above, we can infer that OTUD6B-AS1 can regulate the activity of the Wnt/β-catenin signaling pathway and affect the expression of several EMT-related proteins in ccRCC cells.

### OTUD6B-AS1 inhibits tumorigenesis in ccRCC cells in vivo

**Fig. 7:**
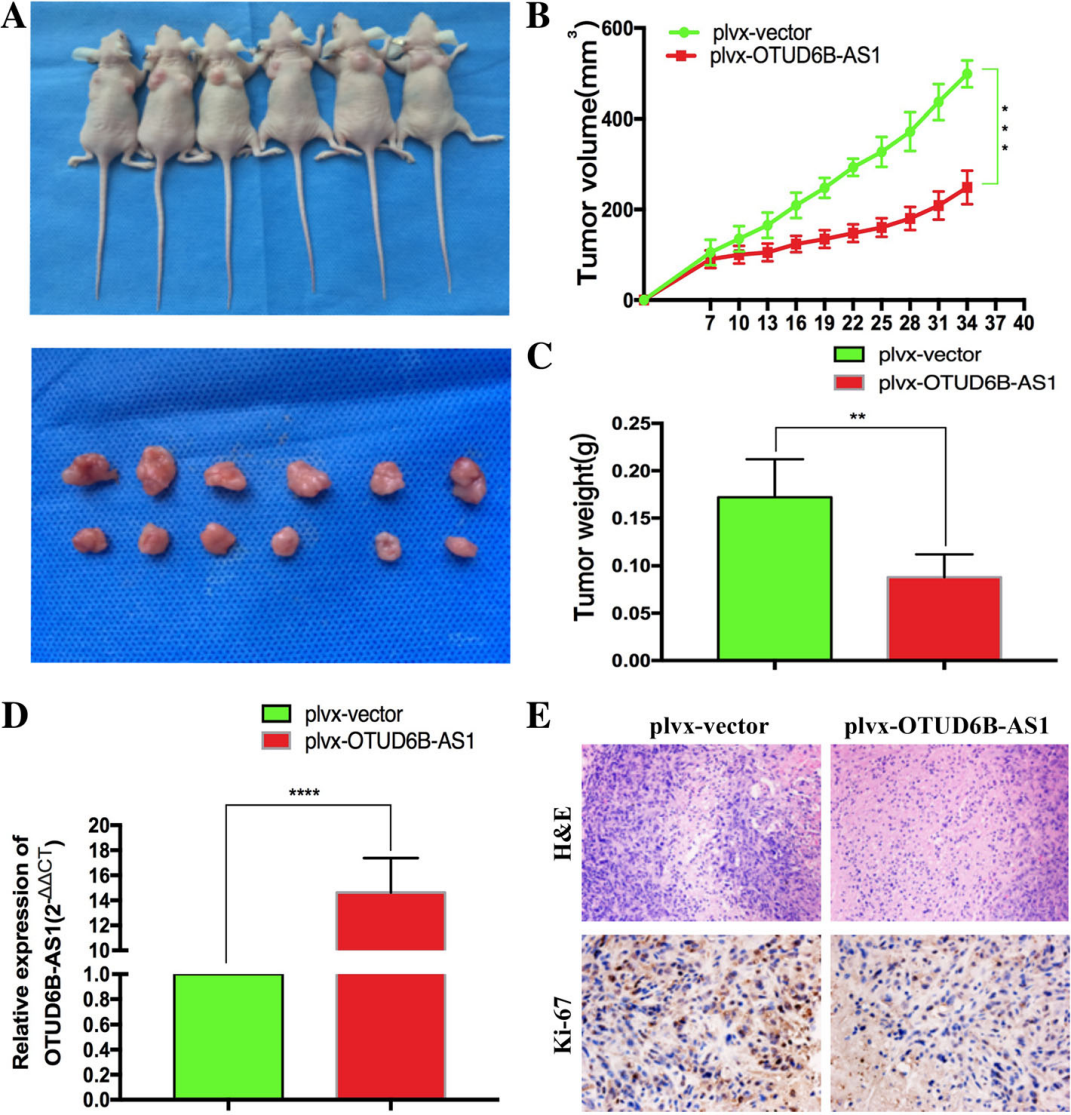
OTUD6B-AS1 inhibited the tumorigenesis of ccRCC cells in vivo. a The empty vector or plvx-OTUD6B-AS1 was transfected into ACHN cells, which were then injected into nude mice (*n* = 6). The tumors formed in the plvx-OTUD6B-AS1 group were dramatically smaller than those formed in the control group. b Tumor volumes were calculated after injection every four days. Points indicate means (*n* = 6); bars indicate SDs. c Tumor weights are represented as the mean tumor weight ± SD. d qRT-PCR was performed to detect the average expression of OTUD6B-AS1 in the xenograft tumors (*n* = 6). e The tumor sections were subjected to H&E staining and IHC staining using antibodies against ki-67. * *P* < 0.05; ** *P* < 0.01; *** *P* < 0.001; **** *P* < 0.0001

To investigate the impact of OTUD6B-AS1 in vivo, we inoculated empty vector- or plvx-OTUD6B-AS1-transfected ACHN cells into nude mice. Compared with the tumors created by the control cells (left), the tumors derived from the plvx-OTUD6B-AS1-transfected ACHN cells (right) were obviously smaller (Fig. 7a and b). Consistently, the tumor weight of the plvx-OTUD6B-AS1 group was remarkably lighter than that of the control group (Fig. 7c). Then, qRT-PCR experiments determined an obvious increase in OTUD6B-AS1 expression in the tumor tissue derived from the plvx-OTUD6B-AS1-transfected ACHN cells compared with that derived from the respective control cells (Fig. 7d). The immunohistochemistry (IHC) results revealed that the tumor tissue derived from the plvx-OTUD6B-AS1-transfected ACHN cells displayed lower ki-67 staining than those formed from the control cells (Fig. 7e). These findings indicate that OTUD6B-AS1 upregulation suppresses ccRCC tumor cell growth in vivo.

## Discussion

In recent research, abnormal expression of lncRNAs has been discovered in various human cancers, and increasing evidence indicates that lncRNAs participate in all steps of carcinogenesis and tumor progression [19–22]. A great number of studies have indicated that lncRNAs play an important role in the diagnosis and treatment of tumors, and lncRNAs can be used as new prognostic markers for tumors [23–26]. The lncRNA OTUD6B-AS1 is oriented in an antisense direction to the protein-coding gene OTUD6B on the opposite DNA strand. Although previous studies have shown that OTUD6B-AS1 expression is deregulated in the skin tissue of patients with systemic sclerosis [27], the mechanism of OTUD6B-AS1 in tumors and other diseases, especially RCC, is unknown. In this study, we identified that the expression of the lncRNA OTUD6B-AS1 was downregulated in ccRCC tissue compared with matched paracancerous tissue, and the downregulation of OTUD6B-AS1 expression was associated with worse clinical characteristics and poor overall survival. We also confirmed the effects of OTUD6B-AS1 on ccRCC cells. The overexpression of OTUD6B-AS1 inhibited the proliferation, migration and invasion of ccRCC cells in vitro and in vivo and promoted apoptosis in ccRCC cells. Collectively, our results demonstrated that OTUD6B-AS1, acting as an antioncogene, may be a potential diagnostic and prognostic biomarker as well as a therapeutic target in ccRCC.

In addition to the abnormal proliferation and differentiation, the invasion and migration of tumor cells also play crucial roles in the process of tumor progression, and EMT is one of the main pathways that regulates invasion and migration. The Wnt signaling pathway, which participates in cell proliferation and regeneration and plays an important role in the development of embryos and the process of EMT, is a highly conserved signaling pathway that is composed of a series of oncogene and antioncogene proteins [28]. The most classical pathway is the Wnt/β-catenin signaling pathway. β-Catenin can combine with E-cadherin to form an E-cadherin/β-catenin complex to maintain the stability of intercellular adhesion structures and cell polarity. The Wnt signaling pathway can inhibit the phosphorylation of GSK3β and the degradation of β-catenin in the cytoplasm and can upregulate the expression of transcription factors, such as Snail and twist, thus inhibiting E-cadherin transcription and increasing the intracellular free β-catenin level, which can activate and stabilize EMT transcription factors in the nucleus and induce EMT. In normal mature cells, the Wnt signaling pathway is inactive, but there is abnormal activation of the Wnt signaling pathway in a variety of tumors, and the inhibition of the Wnt/β-catenin signaling pathway can reduce invasion, metastasis and the occurrence of drug resistance [29–32]. For example, the abnormal activation of the Wnt/β-catenin signaling pathway in liver cancer can promote the malignant transformation of normal hepatocytes [33]; there is also abnormal activation of the Wnt signaling pathway and accumulation of β-catenin in colorectal cancer, and the proliferation of colon cancer cells can be inhibited by inhibiting the activity of the Wnt signaling pathway [34–36]. In different tumors, the role of the Wnt/β-catenin signaling pathway varies. For example, in tongue squamous cell carcinoma, Sox8 can combine with the FZD7 promoter region of the Wnt receptor and activate the Wnt/β-catenin signaling pathway regulated by FZD7, thus regulating tumor cell chemosensitivity, stem cell stemness and EMT [37]. As mentioned above, we explored the mechanism by which OTUD6B-AS1 overexpression affects ccRCC by performing experiments to demonstrate that OTUD6B-AS1 decreased the activity of the Wnt/β-catenin pathway and suppressed the expression of EMT-related proteins in ccRCC cells.

In the current work, we evaluated the function of OTUD6B-AS1 in ccRCC progression and found a negative correlation between ccRCC malignancy and OTUD6B-AS1 expression. However, one of the yet undetermined mechanisms not addressed in the current study is whether OTUD6B-AS1 can function as a competitive endogenous RNA (ceRNA) to regulate microRNAs (miRNAs) during ccRCC development. For example, the lncRNA MIAT can competitively bind with miR-29c and modulate the expression of LOXL2 to regulate the progression of ccRCC [38]. LINC01133 can be combined with miR-106a-3p and then affect the expression of APC, thereby regulating gastric cancer progression [39]. Since ceRNAs represent a novel aspect of regulation, especially during tumorigenesis [40], ongoing work is strongly needed to determine whether OTUD6B-AS1 could be a candidate ceRNA during ccRCC progression. Moreover, with the progress of molecular biology technology, gene therapy for tumors is attracting increasing attention, and how to apply our genes to clinical treatment still needs further study. In addition our data were only obtained with cell lines, and that cell line models have several limitations,so there’s still a lot of work to be done to study OTUD6B-AS1 in depth. This is also the focus of our future research.

## Conclusions

In summary, we first investigated the expression pattern of OTUD6B-AS1 in ccRCC tissue and cell lines. OTUD6B-AS1 may be an indicator of poor prognosis in patients with ccRCC. Furthermore, the overexpression of OTUD6B-AS1 significantly decreased proliferation and tumorigenesis while partly inhibiting cell migration and invasion in ccRCC cells in vitro. More importantly, no study has revealed the molecular mechanism and downstream targets of OTUD6B-AS1 until now. Here, we demonstrated that the antioncogenic effect of OTUD6B-AS1 is partly mediated through the inhibition of the activity of the Wnt/β-catenin pathway and the EMT-related pathway. Additional studies are needed to determine whether OTUD6B-AS1 modulates other targets in ccRCC; however, our findings nonetheless provide novel insight into ccRCC pathogenesis as well as a basis for the improvement of individualized treatment for ccRCC patients.

## Additional file

Additional file 1: **Table S1.** lncRNAs significantly associated with the overall survival. **Table S2.** Patient and tumor characteristics of the three RCC subtype cohorts in TCGA. **Table S3.** Comparison of clinical characteristics between low OTUD6B-AS1 group and high OTUD6B-AS1 group in KIRC cohort. **Table S4.** Univariate and multivariate regression analyses for predicting overall survival in KIRC cohort. **Table S5.** Pathway analyses for High OTUD6B-AS1 group and Low OTUD6B-AS1 group in KIRC cohort from GEO data base. **Figure S1.** The relationship between expression level of A and clinicopathological characteristics and survival rate of patients. (A) The Kaplan-Meier plot of the pathology stages of patients with ccRCC (*n* = 523). (B) The Kaplan-Meier plot of the clinical grades of patients with ccRCC (*n* = 516). (C) The survival time of the patients with pathology stage I + II disease (*n* = 36) was longer than that of the patients with pathology stage III-IV disease (*n* = 16). (D) The survival time of the patients with clinical grade I + II disease (*n* = 39) was longer than that of the patients with clinical grade III-IV disease (n = 13). The data represent the mean ± SD of 3 replicates. * *P* < 0.05; ** *P* < 0.01; *** *P* < 0.001; **** *P* < 0.0001. (DOCX 31 kb)

## Abbreviations

ccRCC: Clear cell renal clear cell carcinoma
EMT: Epithelial-mesenchymal transition
KICH: Kidney chromophobe
KIRC: Kidney clear cell carcinoma
KIRP: Kidney papillary
lncRNA: Long noncoding RNA
OTUD6B-AS1: OTUD6B antisense RNA 1
qRT-PCR: Quantitative real-time PCR
RCC: Renal cell carcinoma
TCGA: The Cancer Genome Atlas
