# Supplementary material for: Novel long noncoding RNA OTUD6B-AS1 indicates poor prognosis and inhibits clear cell renal cell carcinoma proliferation via the Wnt/β-catenin signaling pathway
Source: Mol Cancer. 2019 Jan 22;18:15. doi: 10.1186/s12943-019-0942-1 (PMC6341572; doi:10.1186/s12943-019-0942-1)
Supplement: Supplementary file 1 — Table S1. lncRNAs significantly associated with the overall survival. Table S2. Patient and tumor characteristics of the three RCC subtype cohorts in TCGA. Table S3. Comparison of clinical characteristics between low OTUD6B-AS1 group and high OTUD6B-AS1 group in KIRC cohort. Table S4. Univariate and multivariate regression analyses for predicting overall survival in KIRC cohort. Table S5. Pathway analyses for High OTUD6B-AS1 group and Low OTUD6B-AS1 group in KIRC cohort from GEO data base. Figure S1. The relationship between expression level of A and clinicopathological characteristics and survival rate of patients. (A) The Kaplan-Meier plot of the pathology stages of patients with ccRCC (n = 523). (B) The Kaplan-Meier plot of the clinical grades of patients with ccRCC (n = 516). (C) The survival time of the patients with pathology stage I + II disease (n = 36) was longer than that of the patients with pathology stage III-IV disease (n = 16). (D) The survival time of the patients with clinical grade I + II disease (n = 39) was longer than that of the patients with clinical grade III-IV disease (n = 13). The data represent the mean ± SD of 3 replicates. * P < 0.05; ** P < 0.01; *** P < 0.001; **** P < 0.0001. (DOCX 31 kb) [file 12943_2019_942_MOESM1_ESM.docx]

**Additional file 1**

**Table S1** lncRNAs significantly associated with the overall survival

| lncRNA | Cofficent | p-value | FDR |
| --- | --- | --- | --- |
| *OTUD6B-AS1* | ***-0.44405*** | ***0.008578*** | ***0.033861*** |
| AC006129.2 | 0.239687 | 0.012551 | 0.040928 |
| AC084018.1 | 0.290077 | 0.000128 | 0.001926 |
| DHRS4-AS1 | -0.39807 | 0.000336 | 0.003595 |
| FOXD2-AS1 | 1.426243 | 5.18E-05 | 0.001295 |
| LINC00173 | 0.333755 | 0.014387 | 0.044959 |
| LINC00174 | 0.452507 | 0.001717 | 0.012874 |
| LINC00338 | 0.29512 | 0.005531 | 0.024402 |
| MALAT1 | 0.011375 | 0.012173 | 0.040928 |
| OIP5-AS1 | -0.21251 | 0.002565 | 0.01716 |
| PDXDC2P | 0.363749 | 0.004089 | 0.019167 |
| RP11-174G6.5 | 0.850829 | 0.003606 | 0.018032 |
| RP11-293M10.6 | -3.43676 | 0.009708 | 0.035497 |
| RP11-50E11.3 | -2.6676 | 0.002746 | 0.01716 |
| RP11-732M18.3 | -1.98674 | 0.003303 | 0.018032 |
| RP11-73M18.8 | 0.361981 | 1.04E-05 | 0.00039 |
| RP11-815I9.4 | -0.41359 | 0.003414 | 0.018032 |
| RP4-717I23.3 | 0.361901 | 0.000117 | 0.001926 |
| RP5-1180C10.2 | 0.925377 | 7.88E-03 | 0.032831 |
| SNHG3 | 0.219547 | 0.000767 | 0.007193 |
| SPPL2B | 0.533245 | 0.001454 | 0.012118 |
| TPT1-AS1 | 1.213713 | 1.39E-06 | 0.000104 |

P<0.05 was considered statistically significant in all analyses.

**Table S2** Patient and tumor characteristics of the three RCC subtype cohorts in TCGA

| Variable | KIRC | KIRP | KICH |
| --- | --- | --- | --- |
| Sample (n) | 526 | 256 | 64 |
| Median age (year) | 61(26-90) | 62(28-88) | 52(17-86) |
| Median OTUD6B-AS1 | 1579.95(491.35-8312.33) | 1042.26（335.74-3968.37） | 3335.78（945.48-6608.66） |
| Gender |  |  |  |
| Male | 340（64.6%） | 189（73.8%） | 38（59.4%） |
| Female | 186（35.4%） | 67（26.2%） | 26（40.6%） |
| pathologic stage |  |  |  |
| Stage I | 264（50.2%） | 171（66.8%） | 19（29.7%） |
| Stage II | 57（10.8%） | 20（7.8%） | 25（39.1%） |
| Stage III | 123（23.4%） | 50（19.5%） | 14（21.8%） |
| Stage IV | 82（15.6%） | 15（5.9%） | 6（9.4%） |
| Tumor stage |  |  |  |
| T1 | 270（51.3%） | 174（70.0%） | 19（29.7%） |
| T2 | 69（13.1%） | 25（9.8%） | 25（39.0%） |
| T3 | 176（33.5%） | 55（21.5%） | 18（28.1%） |
| T4 | 11（2.1%） | 2（0.7%） | 2（3.2%） |
| Survival status |  |  |  |
| Alive | 361（68.6%） | 221（86.3%） | 55（86.0%） |
| Died | 165（31.4%） | 35（13.7%） | 9（14.0%） |

**Table S3** Comparison of clinical characteristics between low OTUD6B-AS1 group and high OTUD6B-AS1 group in KIRC cohort

|  | Group | | ***p-value*** |
| --- | --- | --- | --- |
|  | Low OTUD6B-AS1 | High OTUD6B-AS1 |  |
| Sample (n) | 263 | 263 |  |
| Age(year) |  |  |  |
| ≤61 | 145(55.1%) | 135(51.3%) | 0.382 |
| ＞61 | 118(44.9%) | 128(48.7%) |  |
| Gender |  |  |  |
| Male | 182(69.2%) | 105(39.9%) | <0.001* |
| Female | 81(40.8%) | 158(60.1%) |  |
| pathologic stage |  |  |  |
| Stage I/ II | 141(53.6%) | 180(68.4%) | <0.001* |
| Stage III/ IV | 122(46.4%) | 83(31.6%) |  |
| Tumor stage |  |  |  |
| T1/2 | 152(57.8%) | 187(71.1%) | 0.001* |
| T3/4 | 111(42.2%) | 76(28.9%) |  |
| Lymph node metastasis |  |  |  |
| N0 | 134(60.0%) | 103(39.2%) | 0.003* |
| N1 | 15(5.7%) | 1(0.4%) |  |
| Distant metastasis |  |  |  |
| M0 | 181(68.8%) | 240(91.3%) | <0.001* |
| M1 | 74(28.1%) | 1(0.4%) |  |
| Tumor grade |  |  |  |
| G1/2 | 94(35.7%) | 145(55.1%) | <0.001* |
| G3/4 | 168(63.9%) | 116(%) |  |
| Survival status |  |  |  |
| Alive | 159(60.5%) | 202(76.8%) | <0.001* |
| Died | 104(39.5%) | 61(23.2%) |  |

**Table S4** Univariate and multivariate regression analyses for predicting overall survival in KIRC cohort

| Variable | Univariate | | Multivariate | |
| --- | --- | --- | --- | --- |
|  | HR (95% CI) | ***p-value*** | HR (95% CI) | ***p-value*** |
| Age | 1.655(1.213-2.259) | 0.001* | 1.506(1.097-2.067) | 0.110 |
| Gender | 0.953(0.694-1.308) | 0.766 | 0.943(0.680-1.307) | 0.724 |
| Tumor grade | 2.439(1.746-3.407) | <0.001* | 1.625(1.132-2.333) | 0.008* |
| pathologic stage | 4.312(3.106-5.988) | <0.001* | 5.171(2.787-9.593) | <0.001* |
| Tumor stage | 3.480(2.545-4.760) | <0.001* | 0.641(0.355-1.158) | 0.140 |
| OTUD6B-AS1 mRNA level | 0.506(0.368-0.695) | <0.001* | 0.632(0.456-0.874) | 0.006* |

*: statistically significant to predict overall survival rate

**Table S5** Pathway analyses for High OTUD6B-AS1 group and Low OTUD6B-AS1 group in KIRC cohort from GEO data base

| KIRC from GSEA 53757 in EGO(72cases) | | | | |
| --- | --- | --- | --- | --- |
|  | High OTUD6B-AS1 | p-val. | Low OTUD6B-AS1 | p-val. |
| 1 | Oxidative phosphorylation | 0.0 | Olfactory Transduction | 0.0 |
| 2 | Parkinsons Disease | 0.0 | Basal Cell Carcinoma | 0.0 |
| 3 | Proteasome | 0.0 | Hedgehog Signaling Pathway | 0.0 |
| 4 | Aminoacyl Trna Biosynthesis | 0.0 | Neuroactive Ligand Receptor Interaction | 0.0 |
| 5 | Huntingtons Disease | 0.0 | HEMATOPOIETIC_CELL_LINEAGE  Hematopoietic Cell Lineage | 0.0011325028 |
| 6 | Peroxisome | 0.0 | ECM Receptor Interaction | 0.0 |
| 7 | Spliceosome | 0.0 | ASTHMA | 0.010638298 |
| 8 | Protein Export | 0.0 | Cell Adhesion Molecules Cams | 0.0021551724 |
| 9 | Alzheimers Disease | 0.0 | Viral Myocarditis | 0.0047281324 |
| 10 | Citrate Cycle TCA Cycle | 0.0 | Dilated Cardiomyopathy | 0.012373453 |
| 11 | Pentose and Glucuronate Interconversion | 0.003717472 | Calcium Signaling Pathway | 0.0020986358 |
| 12 | RNA Degradation | 0.0 | MELANOGENESIS | 0.009922823 |
| 13 | Propanoate Metabolosm | 0.0 | Cytokine Cytokine Receptor Interaction | 0.0010224949 |
| 14 | Amino Sugar and Nucleotide Sugar Metabolosm | 0.0 | Taste Transduction | 0.03373494 |
| 15 | Glycosylphatidylinositol GPI Anchor Biosynthesis | 0.004651163 | Chemokine Signaling Pathway | 0.013612566 |
| 16 | Ubiquitin Mediated Proteolysis | 0.0 | FOCAL_ADHESION  Focal Adhesion | 0.00931677 |
| 17 | Valine Leucine and Isleucine Degradation | 0.0 | ***WNT Signaling Pathway*** | ***0.026652452*** |
| 18 | Lysosome | 0.0 |  |  |
| 19 | Glyoxylate and Dicarboxylate Metabolism | 0.0037037036 |  |  |
| 20 | Porphyrin and Chlorophyll Metabolism | 0.0 |  |  |

**Figure S1** The relationship between expression level of A and clinicopathological characteristics and survival rate of patients. (A) The Kaplan-Meier plot of the pathology stages of patients with ccRCC (n=523). (B) The Kaplan-Meier plot of the clinical grades of patients with ccRCC (n=516). (C) The survival time of the patients with pathology stage I+II disease (n = 36) was longer than that of the patients with pathology stage III-IV disease (n = 16). (D) The survival time of the patients with clinical grade I+II disease (n = 39) was longer than that of the patients with clinical grade III-IV disease (n = 13). The data represent the mean ± SD of 3 replicates. * P<0.05; ** P<0.01; *** P<0.001; **** P<0.0001.
